# The Minimal *k*-Core Problem for Modeling *k*-Assemblies

**DOI:** 10.1186/s13408-015-0027-4

**Published:** 2015-07-14

**Authors:** Cynthia I. Wood, Illya V. Hicks

**Affiliations:** Department of Computational and Applied Mathematics, Rice University, 6100 Main st, Houston, TX 77005 USA

**Keywords:** Cell assembly, Memory, Graph theory, *k*-Assembly, Complexity, *k*-Core

## Abstract

The concept of cell assembly was introduced by Hebb and formalized mathematically by Palm in the framework of graph theory. In the study of associative memory, a cell assembly is a group of neurons that are strongly connected and represent a “concept” of our knowledge. This group is wired in a specific manner such that only a fraction of its neurons will excite the entire assembly. We link the concept of cell assembly to the closure of a minimal *k*-core and study a particular type of cell assembly called *k*-assembly. The goal of this paper is to find all substructures within a network that must be excited in order to activate a *k*-assembly. Through numerical experiments, we confirm that fractions of these important subgroups overlap. To explore the problem, we present a backtracking algorithm to find all minimal *k*-cores of a given undirected graph, which belongs to the class of NP-hard problems. The proposed method is a modification of the Bron and Kerbosch algorithm for finding all cliques of an undirected graph. The results in the tested graphs offer insight in analyzing graph structure and help better understand how concepts are stored.

## Introduction

The brain’s complex networks of neurons have been studied in an effort to understand human cognition and behavior. In parallel, graph theory and combinatorial optimization have focused in understanding the structure and dynamics of networks that arise from a wide spectrum of applications. In this work, we present mathematical techniques that provide insights in network structure. This is important to the study of the brain since it allows us to recognize structures that play key roles in certain fundamental mental processes. In particular, we focus on the relationship between the study of networks and memory.

Network structure and architecture has been studied to understand sociological and biological problems, mostly to identify cohesive subgroups within social and biological networks. The analysis of subgroups within a network serves to identify the most influential elements in a group; and to understand the interactions between members. Although brain networks are extremely complex, they share certain characteristics with social and biological networks. For further discussion, see [1, 2] and [3]. In particular, the study of interactions within a group is important to the study of neuronal networks, since brain connectivity is crucial to process information. For a more detailed discussion as regards the relationship between networks and its applicability to the study of the brain, see [4] and [5]. In this article, we study two specific network structures, namely a clique and a *k*-core, and their potential applications to the study of associative memory.

A clique is a subnetwork in which the actors are more tied to one another than to other members of the network. In terms of the brain, the actors are neurons and the ties between them represent synapses between these neurons. A clique can be seen as a group of neurons that collectively respond to a particular stimuli. The Hebbian theory of learning is often paraphrased as “Cells that fire together wire together” and refers to groups of neurons that fire in synchrony [6]. In other words, events that occur simultaneously are associated in memory. For instance, in a clique it is only necessary to give excitatory input to a fraction of the clique in order to make the entire network fire. In 1949 Luce and Perry introduced the clique model to analyze experimental data [7]. In addition, this model was used to develop a non-rigorous approach toward the study of network cohesion [8]. The clique model has gained popularity for being the perfect cohesive subgroup due to the existing relationship between each one of its members [1]. As a consequence, neural cliques have been used to model computation in the visual cortex [9], differential memory consolidation [10] and to understand episodic experiences in the hippocampus [11]. Nonetheless, the clique model has limitations and leaves out structures that still respond collectively to certain stimuli if there is not a connection between each pair of neurons. Consequently, it is important to consider structures with properties similar to cliques, even if they are not maximally connected, such as the ones introduced by Seidman and Foster [12]. One of these structures is a *k*-core, which is a subgraph with minimum degree greater than or equal to *k*. For more details on models to overcome limitations of cliques see [13] and [14]. Throughout this work we focus on the relationship between *k*-cores and the insights they provide in the study of associative memory.

Memory is a fundamental mental process in the brain. Some of its attributes are to represent concepts and objects in the brain and recall information. In addition, memory is closely connected to the perceptual and learning processes. Donald Hebb in an effort to understand the behavior of the human brain introduced the term “cell assembly”. He defined it as a group of neurons that are strongly connected and represent a “concept” of our knowledge [6]. It refers to a memorized pattern in the auto-associative memory scheme, and according to Hebb’s definition it plays an important role in the structural change of long-term memory. For more details on associative memories as brain models and its storage capacities see [15]. The aforementioned definition can easily describe features of memory and its relations with other processes. Nevertheless, it is not known if the relations described by cell assemblies exist. If they were to be real, then the nodes of a given network could represent portions of a cell assembly, and its connections will describe the flow of activity in the cortex. For further discussion, see [16].

Hebb’s definition of a cell assembly created a gateway to research involving neuroscience and advanced mathematical techniques. Topology has been used to study stimulus reconstruction, and the used representation is close in spirit to Hebb’s cell assembly [17]. Although the mathematical techniques utilized are different, stimulus reconstruction is related to the work presented in this paper since it helps to describe activity patterns of neuronal population during cognition. In addition, dynamical systems have been used to understand how knowledge and events are represented and processed in the brain [18]. This type of work studies the dynamics of cell assemblies and gives mathematical expressions of the hypothetical dynamics of neuronal populations in the cortex.

Until today, there does not exist enough evidence to contradict Hebb’s definition of a cell assembly. From the physiological point of view, the idea requires variable excitatory synapses that obey Hebb’s rule. In other words, the connectivity is enhanced by coincident pre- and postsynaptic activity [19]. However, this specific point of view is difficult to test due to the unavailability of experimental data. Valentino Braitenberg was the first one to give interpretation to the theory of cell assemblies in terms of neuroanatomy and neurophysiology [20]. Most of the ideas presented on Braitenberg’s work have been thoroughly explored and served as the basis of cell assembly theory. For a detailed discussion of the current state of cell assembly theory see [21]. According to Hebb’s definition, a cell assembly represents only one concept in our brain. This implies that there must exist a large number of cell assemblies in order to store all the concepts in the brain, and it is still not possible to identify all of them. For an efficient and reliable statistical method to detect and identify members of an active cell assembly directly as significant spike synchrony patterns see [22]. In an effort to investigate if the cortical network is sufficient to contain all of our concepts Palm formulated the main problem of the theory of cell assemblies. The problem asked for the total number of assemblies of a given network. In theory, it is possible to find all cell assemblies and determine the solution to the problem. However, due to the complexity of the definition; the number of neurons on a brain-sized neuronal network; and the number of connections per neuron, it still may not be possible, in practice, to solve the problem of finding all cell assemblies. Therefore, let us focus on a particular type of cell assembly called a *k*-assembly.

In this work we extend Palm’s graph theoretical approach toward understanding memory. We show a connection between the concept of a cell assembly and the definition of a *k*-core, which allowed us to define a *k*-assembly. We go beyond Palm’s main problem of the theory of cell assemblies that asks for the total number of assemblies at a fixed threshold, to ask for all the substructures whose excitations cause the activation of an entire assembly for a given threshold. We solve the aforementioned problem by finding all minimal *k*-cores of a given undirected graph via a backtracking algorithm. We present complexity results related to *k*-cores to highlight the mathematical difficulty of the problem and provide numerical results to validate the proposed algorithm.

The following section provides the necessary background to understand the mathematical definition of a cell assembly and a *k*-assembly as well as a brief overview of backtracking algorithms. In particular, we discuss the Bron and Kerbosch algorithm whose backtracking structure is the essence of the algorithm proposed to solve our desired problem. The proposed algorithm to find all minimal *k*-cores and its complexity are discussed in the methods section followed by numerical results. Lastly, a discussion of the work introduced in this paper is given.

## Formulation of the Main Problem, Basic Terminology and Background

The goal of this paper is to find all substructures within a graph $G = (V,E)$ that must be excited in order to activate a particular type of cell assembly that will be defined in this section, the *k*-assembly. In the graph *G*, each vertex *v* in the vertex set *V* represents a neuron, and each edge *e* in the edge set *E* represents a connection between two neurons, the *threshold* is denoted as the minimum number of inputs each node receives in order to become excited. Throughout this paper, the threshold value will be fixed to a particular given integer *k*. However, it is of high interest to study the behavior of networks as the value of *k* changes with respect to time.

In this section, the reader will be introduced to basic terminology necessary to link the concepts of cell assembly and *k*-assembly. The purpose of this section is to state definitions that will be referred throughout this article. For a detailed discussion of cell assemblies see [6].

### The Cell Assembly: A Graph Theoretical Approach

In 1981, Palm proposed a mathematical interpretation of Hebbian theory in the framework of graph theory. He gave a mathematical interpretation to the cell assembly. In order to understand Palm’s mathematical definition of a cell assembly, the reader must be introduced to some background definitions.

Given a simple graph $G = (V,E)$ in which each vertex *v* in the vertex set *V* represents a neuron, and each edge *e* in the edge set *E* represents a connection between two neurons, the *threshold* is denoted as the minimum number of inputs each node receives in order to become excited. Throughout this paper, the threshold value will be fixed to a particular given integer *k*. However, it is of high interest to study the behavior of networks as the value of *k* changes with respect to time.

Given a weighted graph $(G, c)$, where the weight $c(u,v)$ represents the strength of the synapses from neuron *u* to neuron *v* for all edges $uv \in E$. For the rest of this paper, we fix the value of $c(u,v) = 1$$\forall uv \in E$.

#### Definition 1

Given $S\subseteq V$ and an integer *k*, a *threshold function*$f_{k}$ is described by

$$f_{k} (S) = \biggl\{ v \in V \Bigm| \sum_{u\in S} c(u,v)\ge k \biggr\} . $$

**Fig. 1:**
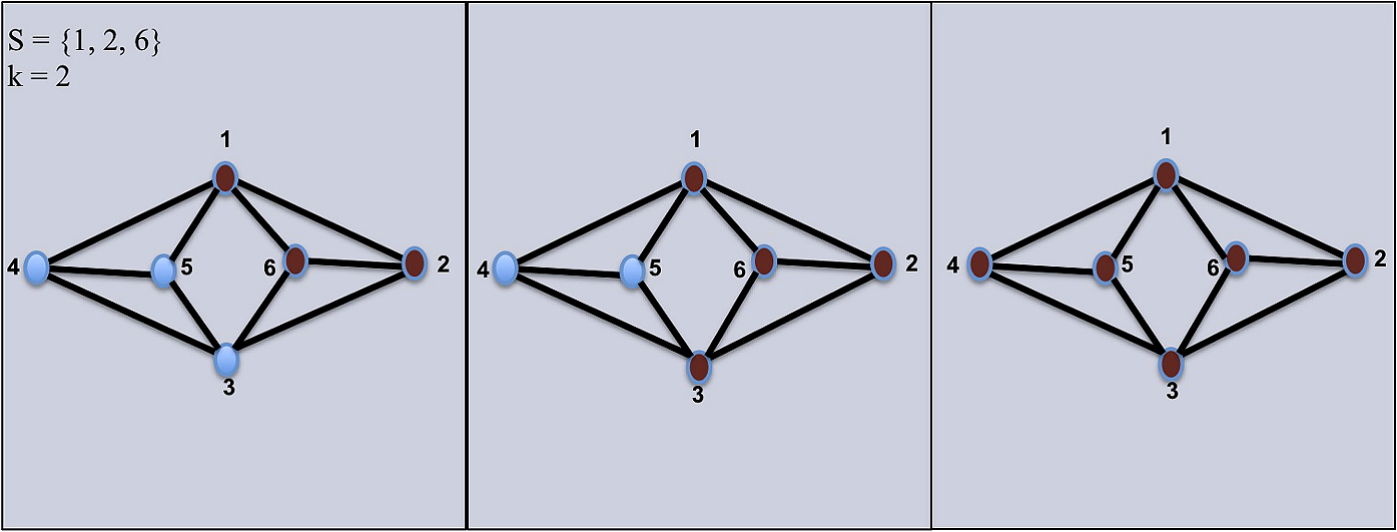
Threshold function $f_{k}$ for $k = 2$. *On the left*, we see the original graph with only the given set $S = \{ 1, 2, 6\}$ excited, $f_{k} (S)$ *in the middle*, and $f_{k}^{2} (S)$ *in the right*

The resulting active set of nodes of $S \subseteq V$ at a threshold *k* is obtained when *S* is given as an input to the threshold function $f_{k}$. That is, given a subset *S* of activated nodes, other nodes in the graph will become activated if they satisfy the threshold inequality, for simplicity we denote $f_{k}^{i} (S) = f_{k}(f_{k}^{i-1}(S))$ for $i \ge2$ and $f^{1}_{k} = f_{k}$. Figure 1 illustrates this process for $k = 2$.

#### Definition 2

A subset of vertices *S* is called *invariant* if $f_{k}(S) = S$.

#### Definition 3

The *closure* of *S*, denoted $\operatorname{cl}_{k}(S)$, is the invariant set generated when $f^{n}_{k}(S) = f^{n-1}_{k}(S)$ for some $n \ge1$.

In Fig. 1, the closure of the set $S = \{1, 2, 6\}$ is achieved when $n = 3$, and it is the entire vertex set *V*.

#### Definition 4

A subset *S* is called *persistent* if $f_{k}(S) \supseteq S$, and it is called *minimal persistent* if no proper subset of it is persistent.

In Fig. 1, the set $S' = \{1, 2, 3, 6\}$ is persistent when $k = 2$. However, $S = \{1, 2, 6\}$ is a persistent subset of $S'$, which implies $S'$ is not minimal.

#### Definition 5

A subset *S* is called *weak* if there exists an $n \ge1$ such that $f_{k}^{n}(S) = \emptyset$.

In Fig. 1, the set $S' = \{ 1, 2\}$ is weak, since $f_{k}(S') = \{ 6\}$ and $f_{k}^{2}(S') = \emptyset$.

#### Definition 6

A *tight* set is a persistent set *P* in which every persistent subset of *P* whose complement in *P* is not weak and excites the whole of *P*.

Finally, the reader has the necessary background concepts to understand Palm’s mathematical definition of a *cell assembly*.

#### Definition 7

A cell assembly (at a threshold *k*) is the closure of the tight set.

The mathematical definition of a cell assembly encompasses a variety of tight sets. For instance, in Fig. 1, *S* is a tight set and any superset of *S* is also a tight set. Yet, Palm proposed that a minimal persistent set is a tight set [19]. Therefore, we focus on the study of cell assemblies generated by minimal persistent sets.

### *k*-Assembly

Seidman introduced *k*-cores to study network structure, and demonstrate that *k*-core cohesion increases as *k* increases [23]. He defined a *k*-core as a maximal connected induced subgraph with degree greater than or equal to *k*. The maximal property of Seidman’s definition will not be considered for the topic presented in this paper. In other words, we define a *k*-core to only be a subgraph with minimum degree at least *k*.

#### Definition 8

A subgraph $K\subseteq G$ is a *k*-core if $| N(v) \cap V(K)|\ge k$$\forall v \in V(K)$.

#### Definition 9

A *k*-core is minimal if no proper subset of its vertices induces a *k*-core.

It is clear by the definition that the subgraph generated by $f_{k}(\tilde{V})$, for some $\tilde{V}\subseteq V$ is a *k*-core if and only if $\tilde{V}$ is a persistent set. That is, if $f_{k}(\tilde {V})$ is a *k*-core, then for all $\tilde{v} \in \tilde{V}|N(\tilde{v}) \cap f_{k}(\tilde{V})|\ge k$, which implies $\tilde{V} \subseteq f_{k}(\tilde{V}) $. Likewise, if $\tilde{V}$ is a persistent set, then $\tilde{V} \subseteq f_{k}(\tilde{V})$, which implies $f_{k}(\tilde{V})$ is a *k*-core. In addition, note that for an unweighted graph, the threshold function definition of a tight set *S* becomes $f_{k} (S) = \{ v \in V \mid | N(v) \cap S |\ge k \}$, that is $\operatorname{cl}(S)$ generates a *k*-core. By definition, a *k*-core is tight as long as its complement is not weak, since every subset of its vertex set is persistent. Hence, the closure of any *k*-core generates a cell assembly.

The definition of a cell assembly tells us that it only takes a fraction of the assembly to get excited in order to excite the entire assembly. However, the motivation and focus of our work comes from the study of cell assemblies generated by tight sets that are minimal, that is, the deletion of any node from the set generates a subset that is not tight. In addition, the mathematical definition of a cell assembly for its study on simple graphs follows the definition of a *k*-core. According to Palm’s definition of a tight set, a particular type of tight set is a minimal *k*-core. Hence, the vertex set of a minimal *k*-core generates a particular type of cell assembly called *k*-assembly.

#### Definition 10

A *k*-assembly is the closure of a minimal *k*-core.

**Fig. 2:**
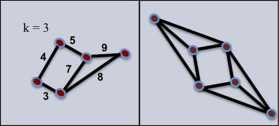
Cell assembly vs. *k*-assembly for $k = 3$. *The graph on the left* satisfies the definition of a cell assembly, but not of *k*-assembly. *The graph on the right* is a 3-assembly with $c(u,v) = 1$ for all $e = uv \in E$

Recall this definition only holds for cases in which the *G* has $c(u,v) = 1$ for all $e = uv \in E$. In Fig. 2, we observe on the left that any two adjacent vertices satisfy the definition of a cell assembly for $k = 3$, since the edges have weights with value greater than one. Nevertheless, a set with less than $k+1$ vertices cannot be a minimal *k*-core, and its closure is not a *k*-assembly. In contrast, the graph on the right has every edge with weight equal to one, and the entire vertex set constitutes a 3-assembly.

The definition of a *k*-assembly and a cell assembly served as motivation to solve the problem of finding all cell assemblies and tight sets that generate them, in particular minimal *k*-cores. The remainder of this paper focuses on solving the problem of finding all minimal *k*-cores for a given simple undirected graph. Nevertheless, finding *k*-cores is not an easy task, and we briefly discuss some complexity properties of problems that deal with *k*-cores in the rest of this section.

#### Theorem 1

*Thek*-*core containment problem is NP*-*complete*.

#### Proof of Theorem 1

The decision version of the problem is the following:

*Instance*: Given a graph *G* and integers $s \le|V|$.

*Question*: Does *G* have a *k*-core of size *s*?

Clearly, the *k*-core problem belongs to NP since given a solution of the problem, a nondeterministic Turing Machine checks if the choice is true in polynomial time. Furthermore, If we restrict the *k*-core problem by considering only instances in which the cardinality of the *k*-core $s = k + 1$, then we get the clique problem [24]. Hence, the *k*-core containment problem is NP-complete. □

The problem of finding all minimal *k*-cores also requires graphical enumeration which refers to the art of counting the number of graphs with a specific property. Note that for some problems to count the number of graphs with a given property is harder than to determine if there exists a graph that satisfies such a property. For instance, “Given a graph *G* and a fixed value $k > 0$, how many distinct *k*-cores are there for *G*?” is not a trivial problem and it empirically depends on the density of the graph. Enumeration problems associated with NP-complete problems are NP-hard [24]. This is true since the enumeration version of the problem must be at least as hard as the decision version of the problem. Hence the enumeration of *k*-cores is NP-hard.

To study in depth enumeration problems the class #*P* was introduced [25].

#### Definition 11

The class #*P* contains all problems computed by nondeterministic polynomial time Turing machines that have the additional facility of outputting the number of accepting computations.

Moreover, #*P*-complete is the analog definition of NP-complete for *P*. The class #*P* asks for the number of solutions rather than their existence. For NP-complete problems counting the number of solutions is #*P*-complete. Therefore enumeration of *k*-cores belongs to the class of #*P*-complete problems.

The detection of minimal *k*-cores is important since they denote the structural motifs (i.e. building blocks of more complex networks) that must be excited in order to propagate the excitation in the graph. The idea of *k*-assembly is related to motif detection [26]. However, instead of restricting it to the study of motifs of certain size, it focuses on the study of subgraphs that pass a certain threshold.

## Previous Work on Solving the Minimal *k*-Core Enumeration Problem

The fact that a clique with vertex set cardinality $k+1$ is a minimal *k*-core allows us to say that algorithms performing clique enumeration were the first ones to attack a subset of the problem we present in this paper.

The Maximal Clique Enumeration Problem (MCEP) asks to compile a list of all maximal cliques in a given undirected graph *G*. Besides its applications in sociological problems, it is also useful in the study of biological networks [27]. MCEP in the worst case scenario runs exponential with respect to the number of vertices. More specifically, the maximum number of maximal cliques in an n vertex graph is $3^{\frac{n}{3}}$ [28]. In other words, it has been proved that there may be a graph with an exponential number of maximal cliques, which implies that any algorithm that solves MCEP for an arbitrary given graph would be exponential.

Bron and Kerbosch (B&K) developed a backtracking algorithm to solve MCEP in 1973 [29]. Although other algorithms to solve the problem were developed around the same period [30], the B&K approach is still one of the most widely known to solve this problem and it is used as a basis for other algorithms that solve MCEP. For further discussion of modifications of B&K, see [31]. The B&K algorithm depends on the number of nodes in the graph, and numerical experiments show it runs in $O (3.14^{\frac{n}{3}})$ on Moon–Mooser graphs with a theoretical limit of $3^{\frac{n}{3}}$. The B&K Algorithm will be discussed in more detail in the following section.

As MCEP, the Minimal *k*-core Enumeration Problem (MKEP) asks to create a list of all minimal *k*-cores in a given undirected graph. There is not a known bound for the maximum number of minimal *k*-cores on a given graph. However, the fact that a clique with vertex set cardinality $k+1$ is a minimal *k*-core, intuitively tells us that the number of minimal *k*-cores grows exponentially in the worst case scenario.

A solution to MKEP through exhaustive search has been proposed, it follows the structure of a branching algorithm [32]. Their algorithm, as well as the one we propose in our methods section initially obtain the maximum *k*-core. The following greedy algorithm obtains the maximum *k*-core in polynomial time [13]:

### Algorithm 1

(Maximum *k*-core)

- $\operatorname{MaximumKcore}(G)$
- **if***G* is empty
  0. **End**
- **else**
  1. Choose a vertex *v*
    of minimum degree $\delta(v)$
  - **if**$\delta(v) \ge k$
    2. The minimum *k*-core is found
    3. **End**
  - **if**$\delta(v) < k$
    4. $\operatorname{MaximumKcore}(G := G\setminus v)$

A description of the algorithm proposed by [32] is the following:

### Algorithm 2

(*k*-Core enumeration of *G*)

- Given an undirected graph $G = ( V, E) $
- 0. **if***G* is a minimal *k*-core
  - **End**
- **else**
  1. Find the maximum *k*-core, call it *H*
- **for** each $v \in V(H)$
  2. $V(G) := V(H) \setminus v$
  3. Go to step 1

The algorithm described above finds all minimal *k*-cores of a given graph. However, a major disadvantage is the fact that it may return the same minimal *k*-core multiple times. It initially checks if the given graph *G* is a minimal *k*-core, and stops in the case it is in fact a minimal *k*-core. Otherwise, it proceeds to find the maximum *k*-core, and then minimal *k*-cores. No numerical results are given for the *k*-core enumeration approach performance. Yet, it is mentioned that it takes minutes to enumerate the *k*-cores of a graph with a vertex set of 10 nodes. The algorithm we developed to solve MKEP will be discussed in our methods section and its performance is analyzed in the numerical results section.

## Methods: Backtracking Algorithm Techniques

Backtracking is a type of recursive strategy commonly used to find all the solutions of some problem. It incrementally builds a tree in a such a way that it faces a number of options at each level, and tries all of them. In a problem with *N* possible solutions, exhaustive search techniques evaluate all the options in *N* trials. In contrast, a backtracking algorithm yields the solution with less than *N* trials, and its solution space is organized as a tree. Initially, it starts at the root of the tree and proceeds to make a choice between one of its children, then it continues to make a choice among the children of each node until it reaches a leaf. Each leaf is either a solution of the problem or does not lead to a solution, and at that point the algorithm backtracks. For more details on backtracking algorithms, see [33] and [34].

In the remaining of this section, we discuss two backtracking algorithms. The first one solves the problem of finding all maximal cliques in a given graph. The second one offers a solution to the problem of listing all minimal *k*-cores of a graph. In addition, an example of a backtracking tree is shown to illustrate the second presented algorithm.

### The Bron and Kerbosch Algorithm for Finding All Cliques of an Undirected Graph

The B&K algorithm utilizes a recursively defined extension operator that is applied to three sets: *compsub*, *not*, and *candidates*. The set *compsub* contains the nodes already defined as part of the clique and it is initially empty. The set *candidates* is the set of nodes adjacent to all nodes in the set *compsub*. The set *not* stores the nodes that had already been processed, leading to a valid extension of the set *compsub* and should remain ignored. In addition to these three sets, there are nodes that are not considered at each step.

In order to obtain all maximal cliques, a backtrack search tree is constructed through recursive calls to the extension operator. Every time the recursion is called the three main sets are modified. The sets *not* and *candidates* are given to the extension operator as input parameters and are locally defined. In contrast, the set *compsub* is globally defined and behaves like a stack. It is important to point out that if at some point the set *not* contains a vertex that is adjacent to all vertices in *compsub*, then the algorithm backtracks since no further selection of candidates will lead to obtaining a maximal clique from the current configuration of the set *compsub*. The basic mechanism can be described in the following pseudocode:

#### Algorithm 3

(Bron and Kerbosch)

- $\operatorname{Extension}(\mathit{compsub}, \mathit{candidates}, \mathit{not})$
- **if**$\mathit{candidates} = \emptyset$ and $\mathit{not} = \emptyset$
  1. Report *compsub* as a maximal clique
- **else** For each vertex $v \in \mathit{candidates}$:
  2. Select a candidate *s*
  3. Add *s* to *compsub* such that
    new $\mathit{compsub} := \mathit{compsub} \cup s$
  4. Create new sets *candidates* and *not*
    by removing all points not connected
    to *s* and store old sets, that is,
    $\mathit{candidates} := \mathit{candidates} \cap N(s)$
    $\mathit{not} := \mathit{not} \cup N(s)$
  5. $\operatorname{Extension}(\mathit{compsub} , \mathit{candidates} , \mathit{not})$
  6. Upon return, remove *s* from *compsub*
    and add it to *not*
    $\mathit{compsub} := \mathit{compsub} \setminus s$
    $\mathit{not} := \mathit{not} \cup s$
- **End**

A clique is found if and only if the sets *candidates* and *not* are empty. If *not* is not empty then the current configuration of the set *compsub* is not maximal. The algorithm terminates if there is no candidates left or if there is an element in *not* that is connected to all elements in the set *candidates*. If the second condition for termination is met, then the addition of any candidate to *compsub* will not be maximal.

To optimize the algorithm and make it terminate as early as possible, the number of times the extension operator is called must be minimized. To do this, every node in *not* is assigned a counter that indicates to how many candidates a node is not adjacent (or disconnected). We then proceed to pick the node with the smallest number of disconnections and on each step select a candidate not adjacent to this node.

### Algorithm for Finding All Minimal *k*-Cores of an Undirected Graph

The problem of finding all minimal *k*-cores of a given graph is computationally expensive. There exists a variety of algorithms to find all cliques in a given undirected graph. However, the B&K algorithm is commonly used to find all maximal cliques, since numerical experiments support its efficiency. We propose a modification of the B&K algorithm to find all minimal *k*-cores on a given graph.

As in B&K, the algorithm presents a backtracking technique to find all minimal *k*-cores. Three sets are utilized to obtain all minimal *k*-cores recursively, namely *kcore*, *not*, and *candidates*. However, since a *k*-core is a generalization of a clique, and every clique on $k+1$ or more nodes contains a minimal *k*-core, but not every minimal *k*-core is a clique, there are some subtle changes in the definition of our sets. For example, we take into account that, given a connected simple undirected graph, all minimal *k*-cores must be contained in the maximum *k*-core, which can be found in polynomial time (for more details see [13]).

The set *kcore* stores the nodes that are part of a *k*-core and is initially the entire vertex set. The set *candidates* contains the nodes that can be deleted to obtain a minimal *k*-core. The set *not* represents the nodes that had already been processed and cannot be deleted from *kcore*. As in B&K, these three sets are modified by a recursively defined extension operator. The set *kcore* is globally defined, whereas the sets *not* and *candidates* are locally defined and handed as parameters to the extension operator.

We construct our backtracking search tree by recursively calling the extension operator. At the root of the search tree, the number of branches generated is equal to the cardinality of the set *candidates*. Each branch corresponds to removing one vertex from our configuration of the set *kcore*, and creating new sets *candidates* and *not*. The algorithm always selects the vertex of smallest degree one at a time. It continues traversing the search tree on a depth first search approach if there is at least one vertex in the set *candidates* whose deletion leads to obtaining a *k*-core of smaller cardinality and backtracks if the configuration of *kcore* cannot lead to returning a minimal *k*-core. That is, if the set *not* contains vertices that must be deleted in order to obtain a minimal *k*-core then no further calls to the extension operator will lead to a valid configuration of the set *kcore*. Hence, such a branch must not be extended. The basic idea behind the algorithm is the following:

#### Algorithm 4

- 0. Obtain maximum *k*-core
- $\operatorname{Extension} ( \mathit{kcore} , \mathit{not} , \mathit{candidates} )$
- **if**$\mathit{candidates} = \emptyset$ and $\mathit{kcore}\setminus v_{i}$
- does not induce a *k*-core $\forall v_{i} \in \mathit{not}$
  1. Report *kcore* as a minimal *k*-core
- **else** For each vertex $v \in \mathit{candidates}$:
  2. Select a candidate *s* of smallest degree
  3. Remove *s* from *kcore* such that
    $\mathit{kcore} := \mathit{kcore}\setminus s$ and
    $\mathit{candidates} := \mathit{candidates}\setminus s$
  4. Create new sets *candidates* and *not*
    and store old sets, that is,
    $\mathit{candidates} := \mbox{set of all candidates}$
    $v \in V\setminus\mathit{not}$ that still leave a *k*-core
  5. $\operatorname{Extension}(\mathit{kcore}, \mathit{not}, \mathit{candidates})$
  6. $\mathit{not} := \mathit{not} \cup s$
    $\mathit{kcore} := \mathit{kcore} \cup s$
    $\mathit{candidates} := \mathit{candidates}\setminus s$

**Fig. 3:**
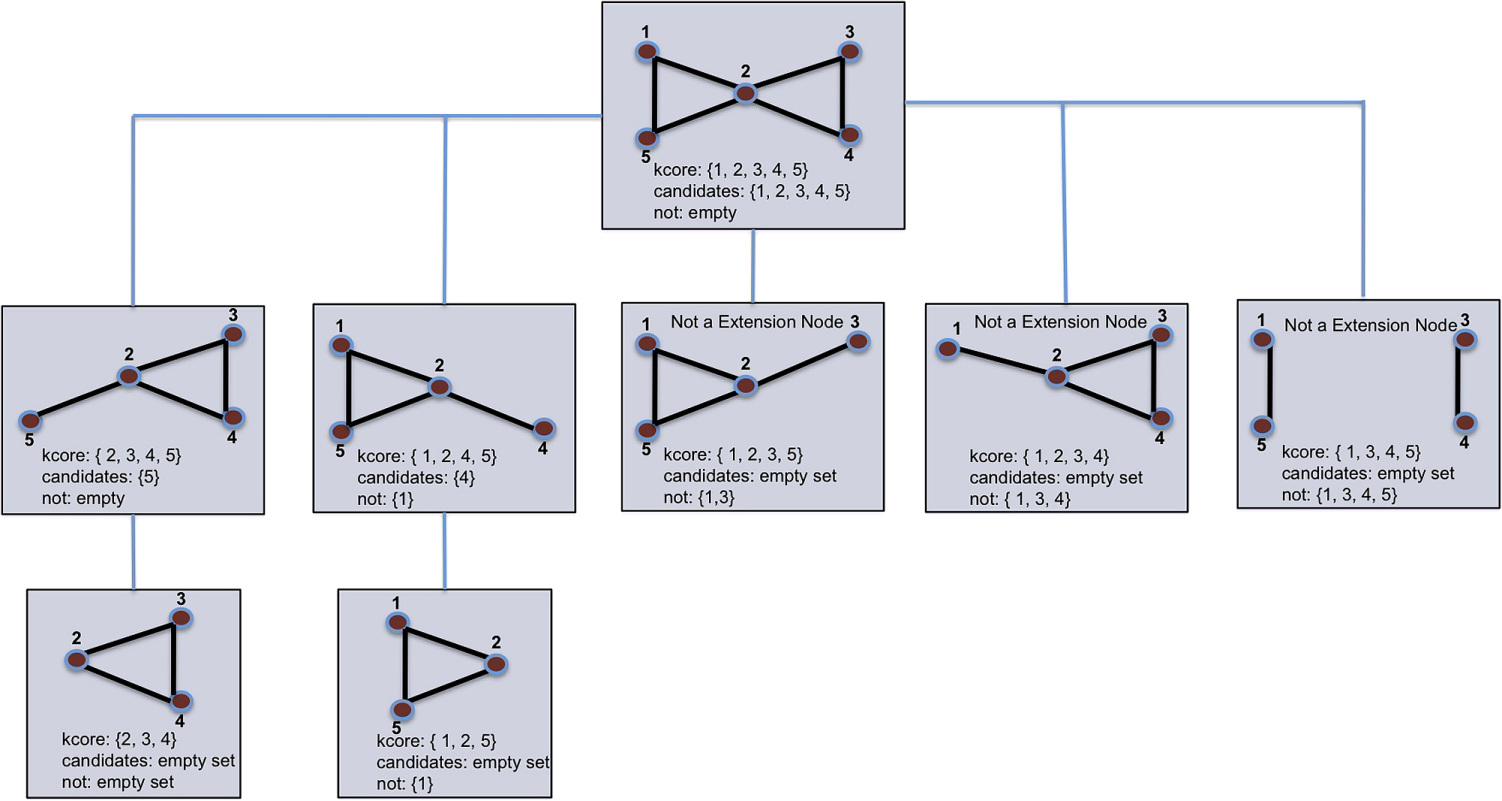
Backtrack search tree for a given graph *G*. The root of the tree contains the entire graph and the leaves contain minimal *k*-cores or extensions that made the algorithm backtrack. The sets *kcore*, *not*, and *candidates* follow the definitions of Algorithm 4

The majority of the steps described above are straightforward to implement. However, there are several different options on how to implement step 2, which is how to select a well-chosen candidate to minimize the number of times the extension operator is called. At the moment, it is impossible to give a good theoretical explanation on why one way to choose a candidate is better than another. They vary on a case by case basis, and its efficiency is determined by observations on numerical experiments. We chose to select a candidate of minimum degree because this way ensures that the set *not* is filled in correctly. However, modifying the original given set of *candidates* to be in the form required to be a candidate and selecting the candidate of maximum degree will also yield a solution to our problem. Figure 3 displays the backtrack search tree of our algorithm for a given graph *G*.

Now, we have to show that our proposed algorithm terminates and performs correctly. Clearly, for a given graph *G* with finite vertex and edge sets the algorithm terminates since the number of subgraphs to enumerate is finite. However, the number of subgraphs in a given graph *G* depends on its structure, and it may be very large for dense graphs. The next theorem is of extreme importance in showing correctness of Algorithm 4, since it guarantees that for every subgraph that contains a *k*-core all its minimal *k*-cores are generated without duplication.

#### Theorem 2

*The extension of the backtracking search tree for a given configuration of the setkcoreby applying the extension operator generates all minimalk*-*cores without repetition that contain*$\mathit{kcore}\setminus v_{i}$$\forall v_{i} \in \mathit{candidates}$

#### Proof of Theorem 2

This proof is by strong induction on the cardinality of the set *kcore*.

For our base case, we consider $|\mathit{kcore}| = k+1$$\forall k > 0$. If $|\mathit{candidates}| = 0$ then *k*-core is minimal. Since we start with the largest *k*-core of the graph and our algorithm only allows one to remove a vertex $v \in V\setminus \mathit{not}$ such that the subgraph obtain by the deletion of this vertex contains a *k*-core. The case $|\mathit{candidates}| > 0$ is not possible, since it implies that there exists a *k*-core of cardinality less than or equal to *k*, which is false by the definition of a *k*-core.

Now suppose that the statement is true for all $l > k \in \mathbb{Z}$ such that $l \le N$, and that all minimal *k*-cores obtained by removing an element of *not* from the current configuration of the set *kcore* have been previously generated. We can suppose the later since it is guaranteed by our definition of the set *candidates*.

Consider a configuration of *kcore* with cardinality $N+1$. Let $\{ v_{1},\ldots, v_{c}\}$ represent the set of candidates for $c\ge0$. If $|\mathit{candidates}\setminus v_{i}| = 0$ for some $0 \le i \le c$ then we see that *kcore* is a minimal *k*-core.

If $|\mathit{candidates}\setminus v_{i}| > 0$, then we have the following two cases:

Choose $\tilde{v}$ as in step 2 of our algorithm and create a new set of $\mathit{candidates} := \mathit{candidates}\setminus\tilde{v}$, proceed to call $\operatorname{extension}(\mathit{kcore}\setminus\tilde{v} , \mathit{candidates} , \mathit{not})$. If the cardinality of the new set of candidates is greater than 0, then by the inductive hypothesis the statement is true for $l = N+1$. If it is zero then $\mathit{kcore}\setminus\tilde{v}$ is not minimal, and $\tilde{v}$ is added to *not*. Which completes our proof and we see that Theorem 2 is true $\forall n \in\mathbb{Z}$. □

Since Theorem 2 is true for any subgraph of any given finite cardinality, we see that Algorithm 4 finds all minimal *k*-cores of a given undirected graph without repetition. In the following section, the results from running the backtracking algorithm for several test instances are presented.

## Numerical Results

Algorithm 4 was implemented using C++ and tested in workstation with a AMD Opteron(tm) Processor 148. Results of numerical experiments are run to test the backtracking algorithm on random graphs. More specifically, we utilized graphs that follow a Bernoulli process in the generation of edges and are known as Bernoulli random graphs, as well as regular graphs. The existence of an edge in Bernoulli random graphs occur independently between each pair of nodes. For instance, given some probability *p* and the number of vertices *n*, there exists an edge $(i,j)$, where $i \neq j$ and $0 < i, j \le n$. In contrast, regular graphs have the property that each vertex has the same degree.

A summary of the obtained results is presented at the end of this section, where 100 Bernoulli random graphs were generated for each test instance, then the average time and number of *k*-cores were computed among the number of graphs that in fact contained at least one *k*-core for $k = 2, 3$ and 5.

The average number of minimal *k*-cores is displayed to highlight the fact that the number of minimal *k*-cores depends on the density of the graph, and not on the value of *k*. Even though every *k*-core is a $k-1$-core, the fact that we restrict our solution set to *k*-cores that are minimal give us cases in which the number of *k*-cores is greater that the number of $k-1$-cores.

The tables below illustrate the performance of Algorithm 4 when the number of vertices $n = 10, 15, 20$ and 25, the probability for generating an edge $p = 0.1, 0.5$ and 0.7 and the value of $k = 2, 3$ and 5. # of *k*-cores denotes the average number of *k*-cores on the tested graphs, and # of graphs is the number of graphs that at least contained one *k*-core.

In Table 1, we observed the results for graphs with 10 vertices. The graphs generated with probability 0.1 only had a few 2-cores, since they are not dense enough to even contain *k*-cores for larger values of *k*. As the probability increased, we observed that more minimal 3-cores and 5-cores were part of the random graphs. However, the average number of minimal 2-cores is always greater than 3-cores and 5-cores. It is important to point out that we observe this behavior only because the vertex set cardinality is small. But it is not always the case that we have more minimal 2-cores than minimal 3-cores as we will see in the later results.

**Table 1:** **Algorithm** **4** **performance for** $\pmb{n = 10}$ **and** $\pmb{p = 0.1, 0.5}$ **and** **0.7**

*k*	*p*	# of *k*-cores	Average time	# of graphs
2	0.1	1.1764	≈ 0 s	17
3	0.1	0	≈ 0 s	0
5	0.1	0	≈ 0 s	0
2	0.5	27.72	≈ 0 s	100
3	0.5	12.2041	≈ 0 s	98
5	0.5	1	≈ 0 s	6
2	0.7	57.14	0.0001 s	100
3	0.7	54.02	≈ 0 s	100
5	0.7	5.4634	≈ 0 s	82

In Table 2, the results for graphs with 15 vertices are displayed. We still observe a low existence of minimal *k*-cores for sparse graphs with $p = 0.1$. However, graphs generated with probabilities 0.5 and 0.7 show a different behavior and contain a larger number of *k*-cores. Note that in contrast to graphs on 10 vertices, on these cases the number of minimal 3-cores is larger than the number of minimal 2-cores and decreases again for the number of 5-cores.

**Table 2:** **Algorithm** **4** **performance for** $\pmb{n = 15}$ **and** $\pmb{p = 0.1}$ **, 0.5 and 0.7**

*k*	*p*	# of *k*-cores	Average time	# of graphs
2	0.1	1.9108	≈ 0 s	56
3	0.1	0	0 s	0
5	0.1	0	0 s	0
2	0.5	166.54	0.0636 s	100
3	0.5	303.01	0.059 s	100
5	0.5	25.22	0.0029 s	90
2	0.7	258.46	0.0577 s	100
3	0.7	630.02	0.0604 s	100
5	0.7	619.09	0.0457 s	100

Table 3 displays the results obtained for random graphs with 20 vertices. In the set of graphs generated with $p = 0.7$, we observe that the average number of minimal 5-cores exceeds the average number of minimal 3-cores and 2-cores. The same behavior is observed in Table 4 for random graphs on 25 vertices with $p = 0.5$ and 0.7. In terms of *k*-assemblies, we observe that at a fixed threshold the number of minimal sets that generate a *k*-assembly increase as *k* increases. This tells us that the number of minimal 2-cores is smaller than the number of minimal 3-cores and 5-cores, which is not true in general if the *k*-cores are not minimal.

**Table 3:** **Algorithm** **4** **performance for** $\pmb{n = 20}$ **and** $\pmb{p = 0.1, 0.5}$ **and 0.7**

*k*	*p*	# of *k*-cores	Average time	# of graphs
2	0.1	6.8370	1.8583 s	92
3	0.1	2	0.485 s	2
5	0.1	0	0 s	0
2	0.5	635.11	2.3256 s	100
3	0.5	3511.19	1.4819 s	100
5	0.5	2661.11	0.0029 s	100
2	0.7	791.66	2.0905 s	100
3	0.7	3902.45	2.3057 s	100
5	0.7	17010.33	2.9131 s	100

**Table 4:** **Algorithm** **4** **performance for** $\pmb{n = 25}$ **and** $\pmb{p = 0.1}$ **, 0.5 and 0.7**

*k*	*p*	# of *k*-cores	Average time	# of graphs
2	0.1	25.13	122.0613 s	99
3	0.1	1.833	2.905 s	6
5	0.1	0	0 s	0
2	0.5	1990.34	85.1718 s	100
3	0.5	25318.58	101.519 s	100
5	0.5	84110.96	117.7972 s	90
2	0.7	1900.64	80.9009 s	100
3	0.7	16796.83	83.4447 s	100
5	0.7	211859.96	109.2354 s	100

In Table 4, we observe an interesting phenomenon, which is that Algorithm 4 finds all minimal *k*-cores of a random graph faster when the graph is dense for the three values of *k* utilized to test it. Although this result may seem counterintuitive, observations showed that the algorithm backtracks faster whenever it is dealing with a dense graph. Algorithm 4 initially takes longer to output the first minimal *k*-core for a dense graph than for a sparse one. However, after the first minimal *k*-core is found; it backtracks to deal with more cases in which minimal *k*-cores in fact exist and with less configurations of the set *compsub* that do not lead to obtaining a minimal *k*-core.

In addition to Bernoulli random graphs, random 5-regular graphs with $n =30$ were tested to check if we observe the same behavior as in random graphs, see Table 5. As expected they only had one minimal 5-core. However, they also contain a greater number of 3-cores than 2-cores.

**Table 5:** **Algorithm** **4** **performance for** **5-regular graphs with** $\pmb{n = 30}$ **and** $\pmb{p = 0.1, 0.5}$ **and 0.7**

*k*	*p*	# of *k*-cores	Average time	# of graphs
2	0.1	29229.97	2512.11 s	100
3	0.1	31860.98	398.71 s	100
5	0.1	1	≈ 0 s	100
2	0.5	29302.06	2512.46 s	100
3	0.5	31907.16	402.9 s	100
5	0.5	1	≈ 0 s	100
2	0.7	29217.7	2511.61 s	100
3	0.7	32036.35	392.18 s	100
5	0.7	1	≈ 0 s	100

The results for the 5-regular graphs are very similar regardless of the probability of their generation. This is due to the fact that they share the same structure. Nonetheless, it is still necessary to check if these types of graphs follow the same behavior as Bernoulli random graphs, since the brain is neither completely random nor regular.

## Discussion

In this paper, we proposed a backtracking algorithm to find all minimal *k*-cores whose excitation can activate a *k*-assembly. The motivation to study this problem emerges from the urge to understand memory. Palm formulated the main problem of the theory of cell assemblies by asking the total number of cell assemblies at a given threshold *k*. The proposed algorithm is closely related to this problem since it allows us to find the total number of subsets that generate *k*-assemblies on a given graph. Through numerical experiments we confirm that fractions of these important subsets overlap. These overlappings tell us that concepts are organized in groups and certain triggers activate associated memories.

An extension to the graph theoretical approach for the analysis of associative memory introduced by Palm is presented along with details on the derivation of the *k*-assembly from the cell assembly model. Although Algorithm 4 is not fast enough to solve the problem in a brain-sized neuronal network, it does offer a solution to the problem, permits us to analyze the structure of a given random graph and gain insight on understanding *k*-assemblies and cell assemblies. For instance, the fact that for some graphs there may be a larger number of minimal 5-cores than 3-cores allowed us to observe how the structures overlap. If we look at it in terms of memory, we can tell that certain nodes are members of several *k*-assemblies, and the absence of one of them may change the structure of the network completely. If larger data sets become available we could use standard techniques for network clustering or *k*-core decomposition that would allows us to partition the graph and find minimal *k*-cores within the partitions.

One of the limitations of our algorithm is that it only finds minimal *k*-cores in undirected graphs and directed graphs are more realistic for real-world applications. However, we can extend the definition of a *k*-core to directed graphs by considering the in and out degree of a given graph. Then we proceed to find minimal *k*-cores in the undirected version of the graph utilizing Algorithm 4. Finally, we check if each of the *k*-cores obtained from the undirected graph is still a *k*-core in terms of in or out degree.

The objective of this project was to gain understanding about the *k*-assembly model and to solve the problem of finding all minimal *k*-cores of an undirected graph. There is still much to explore in the model of the *k*-assembly. In particular, it would be interesting to study the *k*-assembly for a non-fixed value of *k*. For this approach, it would be necessary to analyze the change in the value of *k* with respect to time and design a dynamical system on the graph. In terms of the algorithm, a promising research direction is to explore the structure of the graph to minimize the number of times the extension operator is called; this would be extremely helpful for solving the problem on sparse graphs. In general, the problem of finding all minimal *k*-cores continues to be difficult to solve due to the fact the number of minimal *k*-cores in a graph grows with the number of vertices and edges. Therefore, any condition that makes Algorithm 4 backtrack faster or that minimizes the number of times the extension operator is called would be a significant contribution to the solution of the problem.
